# Differences in Sports Learning by Digital Literacy Level Among Generation Z: An Application of the Unified Theory of Acceptance and Use of Technology (UTAUT) and Media Richness Theory (MRT)

**DOI:** 10.3390/bs16030343

**Published:** 2026-02-28

**Authors:** Kwon-Hyuk Jeong, Chulhwan Choi, Heesu Mun

**Affiliations:** 1 Department of Taekwondo, College of Physical Education, Kyung Hee University, 1732 Deogyeong-daero, Giheung-gu, Yongin-si 17104, Republic of Korea; pure3241@khu.ac.kr; 2 Department of Physical Education, Gachon University, 1342 Seongnam-daero, Sujeong-gu, Seongnam-si 13120, Republic of Korea; 3 Department of Physical Education, Graduate School of Education, Kyung Hee University, 1732 Deogyeong-daero, Giheung-gu, Yongin-si 17104, Republic of Korea

**Keywords:** computer literacy, internet use, virtual reality, technology acceptance, media richness, sports learning

## Abstract

This study examines the differences in sports learning among Generation Z based on digital literacy, using the Unified Theory of Acceptance and Use of Technology (UTAUT) and Media Richness Theory (MRT). As non-face-to-face sports learning—including online lectures, remote coaching, and virtual reality—rapidly expands, digital literacy has become a key factor influencing learning outcomes and equity. Data were collected from Generation Z adults engaged in sports learning through platforms including YouTube, social networking services, online lecture platforms, and mobile applications. Participants were classified into low (*n* = 87)-, medium (*n* = 80)-, and high (*n* = 70)-digital-literacy groups. A 32-item questionnaire adapted from prior studies assessed digital literacy (4 items), four UTAUT constructs (performance expectancy, effort expectancy, social influence, and facilitating conditions; 16 items), and three media richness dimensions (multiple channels, immediacy of feedback, and personalness; 12 items). Confirmatory factor analysis demonstrated acceptable model fit (χ^2^ = 779.013, *df* = 436, *p* < 0.001, NFI = 0.914, IFI = 0.960, TLI = 0.954, CFI = 0.960, SRMR = 0.037, RMSEA = 0.058), reliability (all ω and α > 0.70), and convergent/discriminant validity (all AVE > 0.50; C.R. > 0.70). Group comparisons indicated that higher digital literacy was linked to higher scores in technology acceptance and media richness perceptions (F = 40.364–64.150, *p* < 0.001, *ηp*^2^ = 0.257–0.354) These findings indicate that intra-generational differences in digital literacy shape technology use and media experience in sports learning, highlighting the need to enhance media richness and systematically develop learners’ digital literacy to improve digital sports education’s effectiveness and equity. But causal inferences are limited by the cross-sectional design.

## 1. Introduction

In the field of education, the acceleration of digital transformation has brought significant changes in sports learning as well (Ko, 2020). During the COVID-19 pandemic, restrictions on face-to-face activities accelerated the proliferation of remote education and the development of advanced digital technologies, including artificial intelligence (AI) and the metaverse (C. Wang et al., 2024). As a result, within both the education and sports sectors, non-face-to-face sports learning formats—such as online lectures, remote coaching, and virtual reality (VR) training—have become established as new primary learning formats (Cossich et al., 2023). Consequently, learners who previously relied on home workout videos or fitness applications in a largely passive manner have increasingly engaged more actively in acquiring skills through digital devices, a trend that was further intensified during the pandemic (Mutz et al., 2021).

In recent years, individuals have increasingly recorded and monitored their physical activity using smartphone applications and wearable devices, while also sharing this information on social networking sites to communicate with others (Mutz et al., 2021). In this context, online fitness videos, live-streamed classes, and exercise challenges on social media have evolved into new forms of sports culture mediated by digital media (Frandsen, 2019). During the COVID-19 period, approximately 23% of the total population participated in digital sports activities, such as online fitness programs or home training, and these individuals were, on average, 30 min more physically active per week than those exercised exclusively offline (Mutz et al., 2021). Although these findings suggest that digital technologies can contribute to the maintenance and enhancement of sporting activities, disparities in the utilization and benefits of digital sports content may emerge as a consequence of the digital divide (Goo, 2023). In other words, while individuals in digitally supportive environments are more receptive to online sports learning, those lacking sufficient technological access or proficiency may be unable to fully benefit from emerging digital learning opportunities (Gottschalk & Weise, 2023).

Accordingly, digital literacy—that is, the ability to effectively access, understand, and utilize digital technologies and information—has emerged as a core competency for non-face-to-face sports learning (Özsari et al., 2025). Digital literacy encompasses not only access to information but also the skills required to critically evaluate, integrate, and apply information using digital devices. Gilster (1997) originally defined digital literacy as the ability to understand and appropriately use diverse information acquired via computers for specific purposes. Whereas traditional notions of literacy were largely confined to reading and writing, the concept has expanded in the information age to include the capacity to obtain, process and communicate information through the Internet and information and communication technology (ICT) media (Peng & Yu, 2022). In contemporary societies, where contactless services and digitally mediated interactions have become increasingly normalized, the ability to competently engage with digital technologies is widely regarded as a fundamental life skill (Osler & Zahavi, 2023).

At the policy level, the OECD Learning Compass 2030 presents global trends, including the integration of digital technology as an educational objective and identifies digital literacy as a fundamental competency necessary to transition into future societal structures (OECD, n.d.). Similarly, the “Framework for 21st Century Learning,” developed by the partnership for 21st Century Skills (P21) emphasizes digital literacy, alongside critical thinking, communication, collaboration, and creativity, as an essential core competency for learners in the 21st century (Battelle for Kids, 2019). Reflecting this global consensus, international organizations and national governments have increasingly supported education policies and research initiatives aimed at enhancing digital literacy across diverse learning contexts (OECD, 2019).

Thus, while digital literacy is a crucial element for learners, there is variation in digital literacy levels among learners. Data from the National Information Society Agency (NIA), an affiliate of the Ministry of Science and ICT of the Republic of Korea, indicate that while access to digital information among the general population is very high at 96.5%, levels of digital information utilization at 80.0%, and digital competency remain comparatively lower at 65.6% (Ministry of Science and ICT & National Information Society Agency, 2025). Although Millennials and Generation Z—often characterized as digital natives—generally demonstrate high familiarity with digital devices, considerable intra-generational differences in digital literacy persist (Reid et al., 2023). Digital literacy varies even among members of the same generation depending on educational attainment, exposure to technology, and personal interests. Such differences in ability are likely to influence learning outcomes in digital environments (Stefán et al., 2025).

The rapid advancement of digital technology and proliferation of online learning environments have fundamentally transformed the paradigm of sports education (Deng & Tao, 2025). As digital devices and online learning platforms become increasingly central to sports learning—particularly for younger generations—the level of sports learners’ digital literacy has emerged as a critical determinant of learning effectiveness (Cai et al., 2025; Özsari et al., 2025). Although previous studies have frequently employed path analysis or structural models to examine the effects of digital literacy on learning-related outcomes (E. Y. Park, 2025; Soeprijanto et al., 2022; Zakir et al., 2025), they have not sufficiently examined differences in technology acceptance or media perceptions across varying levels of digital literacy. Moreover, as a significant proportion of existing research has centered on face-to-face sports learning, studies targeting learners who acquire sports skills in digital environments remain inadequate.

Therefore, the present study aims to empirically examine whether differences exist in technology acceptance and media perceptions—based on the Unified Theory of Acceptance and Use of Technology (UTAUT) and Media Richness Theory (MRT)—according to the digital literacy levels of Generation Z sports learners. Specifically, key constructs derived from UTAUT and MRT were treated as dependent variables and group differences were analyzed across levels of digital literacy. By doing so, this study seeks to provide practical foundational data by proposing strategies to apply these findings to sports education within a digital environment.

Furthermore, this study moves beyond a direct application of UTAUT and Media Richness Theory (MRT) by theorizing digital literacy as a boundary condition that shapes how Generation Z learners translate technological affordances into acceptance beliefs (UTAUT) and media experience (MRT). By demonstrating systematic within-cohort heterogeneity (low–medium–high digital literacy) in both UTAUT and MRT perceptions, the study refines prior ‘digital native’ assumptions and offers a theoretically grounded explanation of why identical media environments can yield different perceived richness and acceptance patterns across learners.

Additionally, rather than proposing a new theory, this study aims to extend existing theoretical frameworks by examining how established technology adoption and media perception mechanisms operate within body-centered sports learning contexts. The contribution of this study lies in contextual reinterpretation and boundary specification of UTAUT and Media Richness Theory within intra-generational digital literacy variation.

## 2. Theoretical Background

### 2.1. Digital Literacy in Sports Education

Digital literacy, as conceptualized by Gilster (1997), denotes the ability to use digital tools (such as the Internet and smart devices) to locate information from diverse sources, comprehend and analyze it, critically evaluate its relevance and reliability, and subsequently create, apply, and generate new information and knowledge. Importantly, this concept extends beyond mere usage ability to encompass information utilization capabilities grounded in critical thinking and ethical awareness, thus enabling the assessment of information reliability and value.

Learners’ digital literacy is a significant factor influencing learning satisfaction in environments that use smart devices (Cho et al., 2019). Learners with high digital literacy can proactively utilize digital tools to access, understand, integrate, and evaluate useful information, thereby generating new knowledge (F. Wang et al., 2024). In digital learning environments, learning gaps may arise depending on learners’ digital proficiency (Meng et al., 2024). Research findings indicate that learners with high digital competence tend to be better prepared for online learning, thereby enhancing their self-directed learning abilities (Alanoglu et al., 2025). Furthermore, it was found that higher levels of digital literacy enhance social interaction by enabling smoother communication and collaboration with peers during online classes and improve cognitive engagement through deeper participation in learning content (Getenet et al., 2024).

Digital literacy plays an important role in sports education. To effectively utilize the vast amount of sports information available online effectively, learners must possess sufficient digital literacy. Various digital tools, such as video analysis, wearable devices, and online coaching platforms, have been found to enhance learners’ motivation, engagement, and functional skill acquisition (Martín-Rodríguez & Madrigal-Cerezo, 2025). However, the effective use of such technologies presupposes that learners possess the digital competence necessary to operate and interpret them appropriately. A study targeting university students in the field of sports science reported that higher levels of digital literacy were associated with more positive attitudes toward online learning, with digital competence accounting for approximately 17% of their online learning attitudes (Soyer et al., 2023). Conversely, insufficient digital literacy skills can lead to a digital divide within the sports sector, raising concerns about exclusion from physical activities or learning outcomes facilitated by digital technology (Cai et al., 2025). Consequently, cultivating digital literacy is essential for enhancing learning outcomes and ensuring equitable learning opportunities in contemporary sports education.

In this study, digital literacy is operationalized as a functional learning capability relevant to media-based sports learning rather than as a comprehensive sociotechnical competency framework. This operational scope was intentionally bounded to match the behavioral context under investigation.

### 2.2. Unified Theory of Acceptance and Use of Technology (UTAUT)

With the rapid advancement of information technology, numerous studies and theoretical models concerning user acceptance have emerged. Among these, the most representative are the Technology Acceptance Model (TAM) and the UTAUT. The original TAM aims to explain the critical factors influencing the widespread adoption of computers based on two concepts: perceived usefulness and perceived ease of use (Davis, 1989). Although the TAM has evolved from its initial model, its limitations stem from overly simplified set of variables. Venkatesh et al. (2003) proposed UTAUT as an integrated model of technology acceptance designed to explain individual behavior regarding the adoption of new technologies. The UTAUT is an integrated model of technology acceptance designed to explain individual behavior regarding the adoption of new technologies. Unlike the TAM, which primarily focuses on the functional characteristics of technology, particularly how ease of use influences technology acceptance intentions and behavior, the UTAUT considers social influence, facilitating conditions, and technological support in addition to functional characteristics.

The UTAUT posits that four factors—performance expectancy, effort expectancy, social influence, and facilitating conditions—influence technology acceptance intention and usage behavior. Performance expectancy refers to the extent to which individuals expect their work or learning performance will improve using technology (Alblooshi & Abdul Hamid, 2022). In the context of sports education, this reflects the perceived achievement through digital learning. Effort expectancy refers to the perceived ease and convenience of using a given technology or system and is similar to the concept of perceived ease of use in the TAM (Camilleri, 2024). In sports education, this involves assessing whether learners can easily use digital sports platforms without assistance. Social influence refers to the extent to which individuals perceive that other’s opinions or evaluations affect their intention to use a technology (Hu et al., 2019). For instance, if fellow learners, instructors, or family members positively evaluate or recommend the effectiveness of an online exercise program, learners’ intention to adopt the technology may be strengthened. Finally, facilitating conditions refer to the extent to which users believe that the organizational and technical infrastructure is in place for them to use a particular technology (Ambarwati et al., 2020). In sports learning, enabling conditions include technical support and environment required when using sports apps, wearable devices, or online platforms, alongside tailored education and training for users.

### 2.3. Media Richness Theory (MRT)

MRT, proposed by Daft and Lengel (1986), refers to the degree to which a communication medium enables immediate feedback, conveys multiple cues and channels, supports personalization, and allows the use of diverse language forms. Media richness was initially examined as a factor influencing media use in organizational contexts; however, with advances in ICT, it has gained renewed attention due to its capacity to overcome limitations of traditional media by incorporating nonverbal cues and interactive features (Tang & Geng, 2025). Particularly in sports education and related professional settings, it is essential to move beyond simple instructional methods by utilizing media resources and learning tools alongside diverse media-based instructional strategies. These strategies should incorporate both visual and verbal explanations of movement to ensure that learners can effectively understand and acquire the content. Media richness, which is closely associated with engagement and enjoyment, should therefore be considered in the development of digital sports learning community services (Chen et al., 2025).

Media richness is determined by four criteria: immediacy of feedback, diversity of cues, variety of languages, and personalization. First, immediacy of feedback refers to the extent to which communications enable rapid, real-time interaction (Ferry et al., 2001). In sports learning, this can be illustrated by the contrast between immediate feedback provided during real-time coaching and delayed feedback from prerecorded instructional videos. Second, diversity of cues refers to the range of channels used to convey information, such as visual, auditory, and bodily cues (Daft & Lengel, 1986). In sports education, learning outcomes are enhanced when these cues operate in an integrated manner. For example, visual demonstrations, verbal instructions, and body language cues emphasizing center-of-gravity shifts can deepen learners’ understanding (Penalver-Andres et al., 2021). Third, language variety enhances learner immersion and comprehension through the use of nuanced expressions and sensory-oriented descriptions (Daft & Lengel, 1986). In sports learning, technical skills are often internalized through figurative or experiential language. Finally, personalization refers to the extent to which messages or feedback can be tailored to the learner’s characteristics and situational context (Daft & Lengel, 1986). In sports learning, feedback and learning content are tailored according to the learner’s level, physical condition, and objectives. MRT provides a theoretical framework for examining whether learning outcomes differ depending on the characteristics of the media through which sports learning is delivered.

As presented in these prior studies, Digital literacy as a boundary condition linking UTAUT and MRT. While UTAUT explains technology-acceptance beliefs and MRT explains perceived richness of communication media, both frameworks implicitly assume that users can access and interpret digital cues with sufficient competence. We propose that digital literacy constitutes a personal resource that conditions (1) how efficiently learners form UTAUT beliefs (performance expectancy, effort expectancy, social influence, and facilitating conditions) and (2) how effectively they perceive and utilize richness features (multichannel cues, immediacy of feedback, and personalness). Therefore, the perception of richness in digital sports learning should be understood as varying according to competence. This boundary condition logic provides the basis for proposing a hypothesis comparing groups based on their level of digital literacy.

## 3. Research Hypothesis

### 3.1. Differences in UTAUT According to Digital Literacy Levels in Sports Learning

Digital literacy is the learner’s capacity to navigate, comprehend, and utilize information within digital environments while generating new knowledge. It extends beyond technical proficiency to encompass critical thinking and self-directed learning abilities (Gilster, 1997; Nikou et al., 2022). The recent acceleration of digital transformation in sports education has seen digital literacy levels emerging as key determinants of how effectively learners can adopt and utilize online platforms, fitness applications, and virtual sports content (H. Li et al., 2025; Mutz et al., 2021; Turhan, 2023). Learners with high digital literacy efficiently utilize the diverse features of learning platforms (e.g., repeated learning, video analysis, real-time feedback) and expand their interactive learning experiences through participation in online communities (Z. Li et al., 2022). Learners with high digital literacy perceive digital media not merely as a means of information transmission, but as a tool for knowledge expansion that enhances self-directed learning (Lin et al., 2020; Rasheed et al., 2020). Conversely, learners with low digital literacy experience cognitive strain due to system usability issues, difficulties in information retrieval, and anxiety about technical errors, which are likely to diminish their motivation to learn (Teo, 2019; Chen et al., 2025). Such disparities are not merely a matter of individual ability but may act as structural factors that threaten the efficiency and equity of digital sports education.

Previous studies have also reported that higher levels of digital literacy strengthen positive attitudes toward technology adoption and intentions for continued use (Alshammari & Alkhwaldi, 2025; Kabakus et al., 2025). Particularly within the context of sports learning, learners with high digital literacy have been found to perceive greater performance expectations and ease of use from online training platforms, while also enhancing immersive experiences through feedback and interaction (Özsari et al., 2025; Swim et al., 2023; Turhan, 2023). Accordingly, it is anticipated that differences in technology adoption levels will be significantly evident depending on digital literacy levels, leading us to propose the following hypotheses:

**H1.**  *In sports learning, the UTAUT will vary according to digital literacy levels*.

### 3.2. Differences in MRT According to Digital Literacy Levels in Sports Learning

According to Daft and Lengel (1986), communication media exhibit varying degrees of richness depending on the clarity of information transmission and the reduction in ambiguity. That is, the relationship between digital literacy and MRT is closely linked from the perspectives of ‘information interpretation ability’ and ‘efficiency in media selection’ (T. K. Yu et al., 2017). Therefore, digital literacy is deemed a prerequisite for recognizing media abundance and selecting and utilizing appropriate media.

In prior research, digital literacy and media richness play a significant role in sports learning (Oregon et al., 2018). When learning sports skills, considerable equivocality and uncertainty arise during exercise tasks (Sannes & Kim, 2018). Coaches and instructors convey complex physical movement techniques to learners, making it difficult to effectively communicate the multiple cues learners require through simple text-based communication alone (Z. Wang, 2022). According to MRT, to maximize learning effectiveness when tackling tasks of high complexity, one should select media that provide immediate feedback, convey multiple cues, offer linguistic diversity, and maintain a high degree of personal focus (Oregon et al., 2018). Thus, the extent to which one recognizes and utilizes the richness offered by these media varies significantly, depending on the level of digital literacy (Mammadov, 2022). Generation Z is inherently digitally native generation accustomed to multisensory input and rapid information processing; however, disparities in digital literacy between individuals can lead to significant differences in perceptions of media richness and learning outcomes (Su et al., 2024).

Therefore, it is anticipated that the degree to which variables, such as immediate feedback, multiple cues, linguistic diversity, and personalized focus—which constitute perceptions of media richness in sports learning—manifest will differ according to digital literacy levels. Consequently, we propose the following hypothesis:

**H2.**  *In sports learning, MRT will vary according to digital literacy levels*.

## 4. Materials and Methods

### 4.1. Study Design

This study investigated differences in the UTAUT and MRT across two levels to verify the possibility that differences in digital literacy levels among Generation Z students may lead to variations in sports learning by digital media. A quantitative research design and convenience sampling were employed. Participants were provided with an online questionnaire containing the research objectives and guidelines for participation. Data collection was conducted over a period of two months, beginning in November 2025.

### 4.2. Participants

The survey participants were adults from South Korea who were at least 20 years old and had engaged in sports learning through media within the past year. This included Generation Z members (those born between 1995 and early 2010s). Participants were recruited from university sports clubs and related associations in South Korea, yielding a context-specific sample of Korean Generation Z students involved in media-based sports learning. The primary analytic focus was on intra-generational heterogeneity (low vs. medium vs. high digital literacy) within Generation Z, for which a relatively homogeneous age cohort helps isolate literacy-related differences from age or cohort effects. Accordingly, findings are interpreted as evidence of within-cohort variation in technology acceptance and media experience, rather than as estimates intended to generalize to broader populations (Shadish et al., 2002).

A total of 237 participants were recruited for this study. All participants were members of university-affiliated sports clubs in South Korea, including central sports clubs, departmental sports clubs, and small groups engaged in specific sports activities. Sports clubs, such as futsal, running, and fitness golf, were mostly composed of younger individuals.

This study was approved by an IRB. To conduct the survey, an online questionnaire was used. This included information on the study and participation. Before starting the survey, participants were presented with an online information sheet and were required to provide electronic informed consent by checking an “I agree to participate” box to proceed. The questionnaire was administered to those in charge of these clubs and their associations via QR codes and links. Participants could choose to participate in the study. The participants were not forced to participate in the study. All the information that the people participated in was kept confidential. The survey was completed anonymously (no personally identifying information was collected), and participants were reminded that there were no right or wrong answers to reduce evaluation apprehension and socially desirable responding. This indicates that no one was at a disadvantage when not participating in the study.

### 4.3. Data Collection Tools

To identify the demographic characteristics of the respondents, three questions were assessed sex and highest level of education attained. Additionally, variables related to sports learning were measured, including type of sport participated in, primary sports learning media used, primary purpose of using learning media, and average time spent on sports learning.

Digital literacy was used as an independent variable for comparative analysis. The digital literacy factor was measured as a single factor comprising four items, based on the scale used in Gilster (1997). In this study, digital literacy was operationalized as a parsimonious indicator of functional digital competence relevant to sports learning media use, rather than as a comprehensive multidimensional literacy framework. This operational choice was made to support clear group-based comparisons aligned with the study aim (intra-generational differences within Generation Z) and to maintain measurement brevity within the 28-item instrument. Nevertheless, we acknowledge that digital literacy can be conceptualized as multidimensional, and future research should employ broader multidimensional measures to capture domain-specific facets (e.g., information evaluation, content creation, and safety).

This study used an adapted UTAUT-based measurement instrument for the sports learning media context. Specifically, four core UTAUT determinants of technology acceptance and use were extracted and operationalized based on the original framework proposed by Venkatesh et al. (2003) and the subsequent adaptation by Y. H. Lee and Ryu (2019). The UTAUT comprises four constructs: performance expectancy (PE; four items), effort expectancy (EE four items), social influence (SI four items), and facilitating conditions (FC four items).

Subsequently, based on the theoretical framework first proposed by Ferry et al. (2001) in their MRT, a questionnaire modified and supplemented by H. S. Yu (2025) to analyze learning effects according to lecture type (offline vs. online) was adapted and refined for suitability within the sports learning media environment. The MRT comprised four constructs: multiple channels (MC; four items), immediacy of feedback (FB; four items), and personalness (PS four items).

All survey items employed a seven-point Likert scale ranging from one point (“Not at all”) to seven points (“Very much so”).

### 4.4. Data Analysis

Statistical analyses were conducted using SPSS version 28.0, based on 234 valid responses, following the procedures outlined below. Because all focal constructs were measured via self-report, CFA common latent factor approach. First, frequency analysis was conducted to examine participants’ basic demographic characteristics. Second, confirmatory factor analysis (CFA) was performed to validate the measurement model. Cronbach’s α and McDonald’s ω were calculated to assess internal consistency reliability. Third, descriptive statistical analyses were conducted to calculate the mean, standard deviation, skewness, and kurtosis of the key constructs. During this process, digital literacy scores were verified prior to group classification. Fourth, Pearson’s correlation analysis was conducted to examine relationships among the key variables. Finally, Digital literacy scores were divided into three groups using tertile cut-off points to enable interpretable group comparisons rather than to maximize statistical significance. This grouping strategy was adopted because the aim of the study was to examine intra-generational differences in learning characteristics across literacy levels rather than to estimate predictive relationships using continuous modeling. Control variables were not included because the research objective focused on structural differences within a single generational cohort (Generation Z), where demographic variability is relatively constrained. Including additional controls would shift the interpretation toward predictive modeling rather than comparative interpretation, which was not the purpose of this study. Therefore, A one-way multivariate analysis of variance (MANOVA) was conducted to examine differences in the UTAUT and MRT variables according to digital literacy levels. The significance level for all statistical analyses was set at *p* < 0.05, and post hoc analyses were conducted where appropriate.

## 5. Results

### 5.1. Participants’ Basic Characteristics

The required sample size for this study was determined using the computer program G*Power 3.1.9.7. The primary variable of interest was digital literacy, which was categorized into three groups: high, medium or low. Eight variables were examined to determine the effect of digital literacy. The results indicated that a minimum of 225 participants was required for adequate statistical power. To account for potential dropouts and incomplete responses, 12 additional participants were recruited. In total, 237 questionnaires were collected. After reviewing all collected questionnaires, they were deemed valid and all were included in the analyses.

Before analyzing the data, participants’ digital literacy levels were assessed. Scores were divided into three groups: high, medium, and low. Respondents were ranked according to their total digital literacy scores. The 33.3rd and 66.7th percentile values were then used as cut-off points to categorize participants using the tertile method. Respondents with digital literacy scores in the bottom 33.3% were classified as the low group, between 33.3% and 66.7% were classified into the medium group, and those in the top 66.7% or higher were classified into the high group. This three-quartile-based grouping ensures relatively balanced sample sizes across groups, providing sufficient statistical power and stability for group comparison analyses (MacCallum et al., 2002). Furthermore, compared with mean split or extreme group designs, the tertile classification method distinguishes respondents’ relative digital literacy levels while minimizing sample loss (Preacher et al., 2005).

The distribution score cut-off for the low group at the 33.3% percentile was 4.5, comprising 87 individuals; the cut-off for the middle group at the 66.7% percentile was 5.5, comprising 80 individuals; and the high group comprised 70 individuals with scores exceeding 5.5.

**Figure 1:**
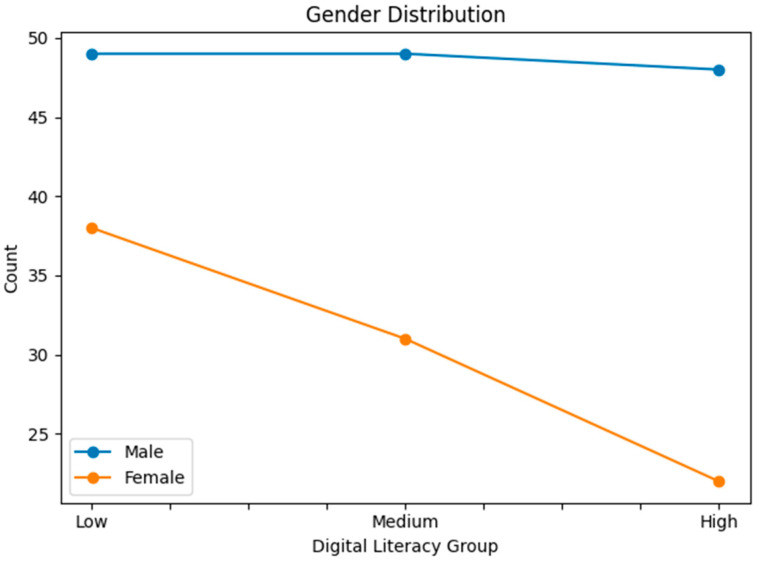
Gender distribution across digital literacy groups.

**Figure 2:**
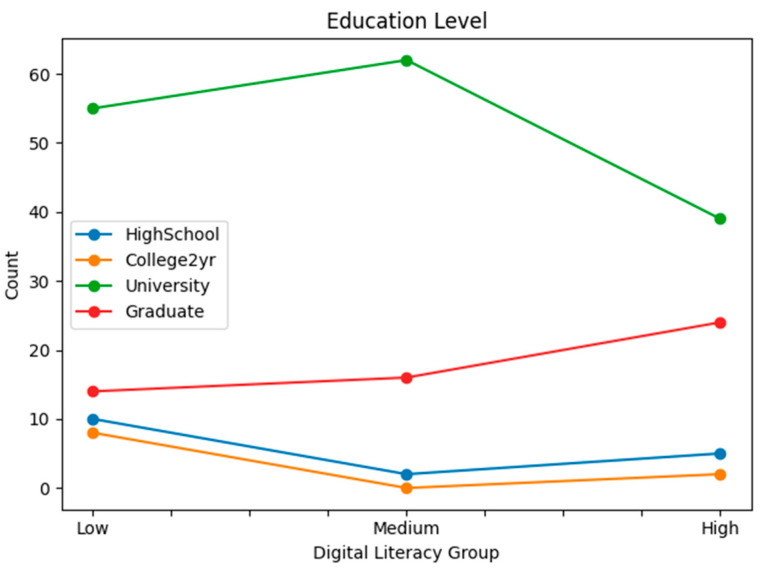
Education level by digital literacy group.

**Figure 3:**
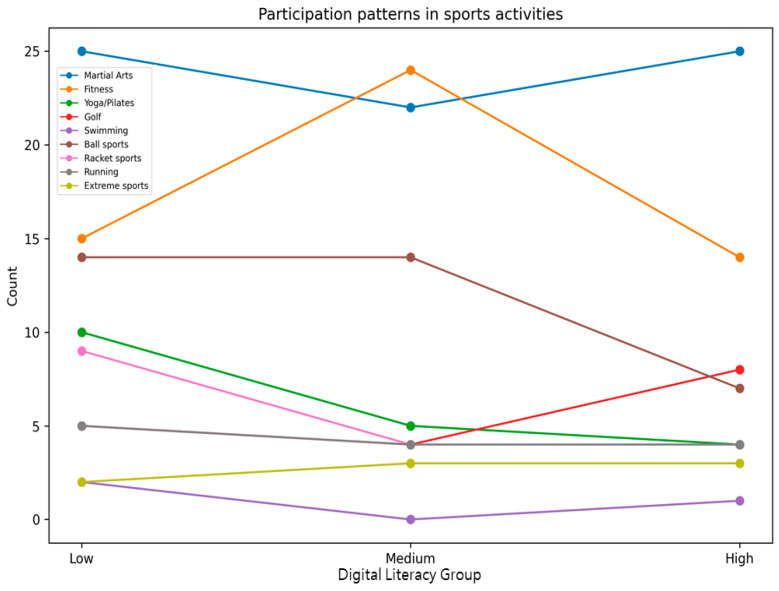
Participation patterns in sports activities by digital literacy group.

**Figure 4:**
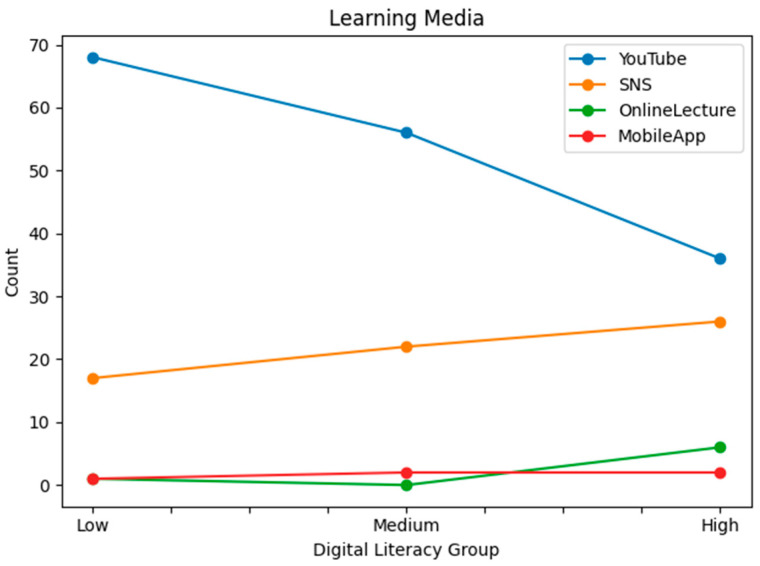
Primary sport learning media by digital literacy group.

**Figure 5:**
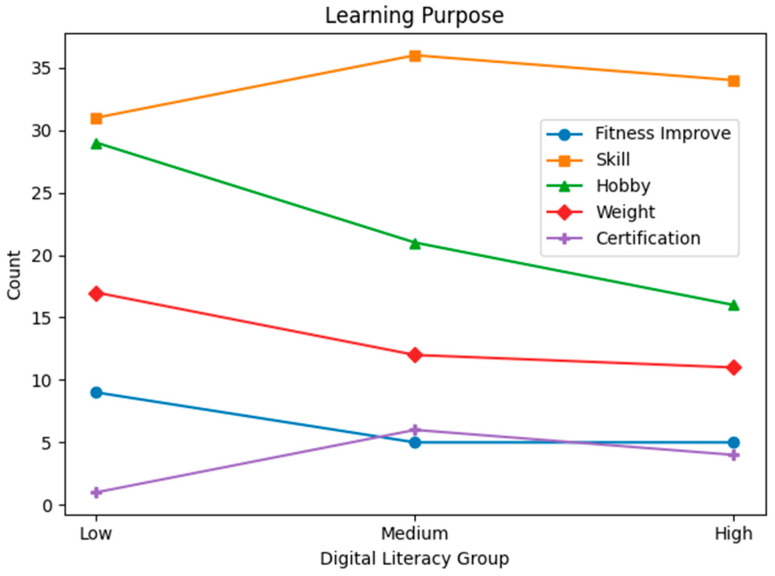
Main purpose of learning media use by digital literacy group.

**Figure 6:**
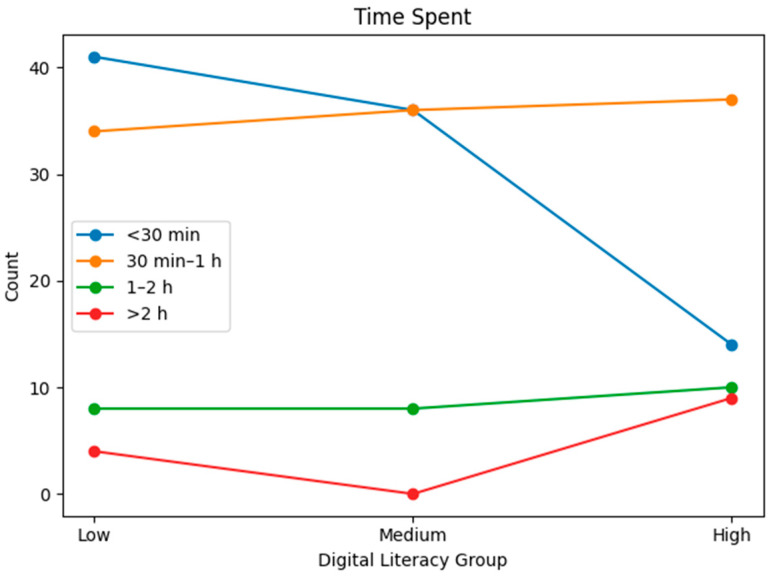
Average time spent on sports learning media by digital literacy group.

Statistical analyses were conducted for each group based on gender, generation, education level, main sports learning discipline, sports learning media, main purpose of sports learning media, main learning devices used, and the time spent on sports learning media. Table 1 presents demographic characteristics by digital literacy level. To complement the tabular description, line-based visualizations were added (Figure 1, Figure 2, Figure 3, Figure 4, Figure 5 and Figure 6).

### 5.2. Scale Validity and Reliability

The questionnaire consisted of 32 items designed to measure eight observed variables, excluding demographic characteristics: a single factor, digital literacy (DL); four UTAUT components (Performance expectancy, Effort expectancy, social expectancy, and Social influence); and three MRT components (Multiple channels, Immediacy of feedback, and Personalness). Responses were evaluated on a 7-point Likert scale. The independent variable, digital literacy, was measured using four items employed by Gilster (1997). The dependent variable, UTAUT, was measured using 16 items (four sub-factors) employed by Venkatesh et al. (2003), while MRT was measured using 12 items (three sub-factors) employed by Ferry et al. (2001).

Experts in the relevant field verified the content validity of the questionnaire used in this study. Subsequently, CFA was conducted to establish convergent and discriminant validity. The CFA results showed that the fit indices of the measurement model met the criteria for structural models proposed by Kline (1998) (CFI > 0.90, IFI > 0.90, TLI > 0.90, RMSEA < 0.10, SRMR < 0.05), as shown in Table 2, confirming the acceptability of the analysis results.

In addition, to examine whether the covariance among the observed variables could be attributed to a single measurement source, a one-factor CFA model in which all items loaded onto one latent factor was estimated and compared with the hypothesized multi-factor measurement model. The one-factor model showed substantially poorer fit than the proposed measurement model, indicating that the relationships among the variables were unlikely to be explained by a single common method factor.

To analyze the convergent validity of each variable used in this study, we calculated the construct reliability (CR) and average variance extracted (AVE) values. The CR of all the observed variables used in this study ranged from 0.861 to 0.916, and the AVE ranged from 0.607 to 0.732. These values met the criteria proposed by Anderson and Gerbing (1988) and Fornell and Larcker (1981) of CR ≥ 0.7 and AVE ≥ 0.5, confirming convergent validity of all variables. Furthermore, when comparing the AVE of the construct concepts with the square correlation coefficients between these concepts to verify discriminant validity, the AVE was found to be higher than the square correlation coefficients, thus confirming discriminant validity among the constructs (see Table 3). The reliability analysis of the eight construct concepts used in this study showed that all the variables exceeded the benchmark value of 0.70, as recommended by Nunnally and Bernstein (1994) and McDonald (1999), thereby ensuring the reliability of the items for each factor (ω = 0.921–0.965; α = 0.920–0.965; see Table 3).

### 5.3. Multivariate Analysis of Variance (MANOVA)

The following assumptions were verified before the analysis: (a) independence, (b) random sampling, and (c) homogeneity of the covariance matrices. Randomly selected respondents completed the survey independently, without any overlap. Box’s test was performed to verify the homogeneity of the covariance matrix. This study comprised three groups, each with an equal sample size. This means that the size of one group should not exceed 1.5 times that of the other.

We applied MANOVA to verify the differences between the UTAUT and MRT according to the digital literacy groups. First, the analysis of covariance homogeneity revealed a statistically significant difference (Box’s M = 86.294, F = 1.475, *p* < 0.12). Furthermore, statistically significant differences were confirmed among the three groups (Wilks’ Lambda = 0.458, F = 15.549, *p* < 0.001, *ηp*^2^ = 0.323). The magnitude of effects was interpreted using conventional benchmarks for partial eta squared (*ηp*^2^), where 0.01, 0.06, and 0.14 indicate small, medium, and large effects, respectively (Richardson, 2011). In accordance with the findings of the present study, the observed multivariate effect (*ηp*^2^ = 0.257–0.354) is indicative of a substantial effect. The analysis revealed statistically significant mean differences across all the factors: (a) Performance expectancy, (b) Effort expectancy, (c) Social influence, (d) Facilitating conditions, (e) Multiple channels, (f) Immediacy of feedback, and (g) Personalness.

Post hoc tests were conducted to determine which of the three groups showed significant differences. The results showed that across all factors, the group with high digital literacy (G3) obtained the highest average scores, followed by the intermediate group (G2), and the low group (G1). Detailed results from the MANOVA (Table 4), group-specific means (Table 5), and post hoc analyses (Table 6) are reported below.

## 6. Discussion

This study analyzed group differences in the effects of the UTAUT factors and MRT perceptions on sports learning environments according to Generation Z’s digital literacy levels. The study found that groups with higher digital literacy levels scored higher across all variables, suggesting that even within the technology-savvy generation, polarization in adoption exists based on proficiency levels. Therefore, the research findings should be interpreted as relative and comparative tendencies among literacy groups, and it is important to note that they do not represent precise causal or predictive effects.

### 6.1. Theoretical Implications

This indicates that learners with strong digital competencies perceive new sports technologies as useful learning tools, actively embrace them, and respond positively to systematic support. These results support the original theory proposed by Venkatesh et al. (2003), while suggesting that within generations familiar with digital environments, the ‘digital divide’ based on individual literacy levels creates differences in the psychological mechanisms of technology adoption. The present findings should be interpreted not only as empirical differences but as a boundary validation of the original UTAUT assumptions. Venkatesh et al. (2003) conceptualized performance expectancy and effort expectancy as cognitive evaluations formed under relatively stable technological competence. Our results suggest that within a single age cohort, variation in digital competence restructures these belief formations, implying that user capability functions as a conditioning layer rather than merely an external moderator. In particular, this aligns with the findings of the preceding study by Martins et al. (2014), which indicated that as users’ proficiency increases, barriers to technology use decrease and the sense of accomplishment is maximized. Additionally, unlike prior adoption studies treating literacy as an external antecedent variable, the present results indicate that literacy reorganizes the internal structure of UTAUT belief formation (Ke et al., 2025; Liu et al., 2025). This aligns with recent arguments that technology acceptance models require competence-sensitive interpretation in experiential learning domains rather than purely utilitarian systems (Al-Adwan et al., 2023; Rosli & Saleh, 2023).

Second, from the perspective of MRT, the high-literacy group perceived multiple channels, immediacy of feedback, and personalness more strongly. This indicates that learners proficient in using digital tools are better able to integrate the visual and auditory data (multichannel) provided during sports learning and optimize (personalize) the real-time data (immediate feedback) obtained through AI or wearable devices to suit their own performance capabilities. In other words, learners with higher digital proficiency can process complex media cues such as VR, augmented reality (AR), and wearable data as rich learning resources rather than as information overload.

These results suggest that the information delivery capability of media, as proposed by Daft and Lengel (1986), may vary depending on learners’ receptivity. This supports the original premise of Media Richness Theory (Daft & Lengel, 1986) that communication effectiveness depends on uncertainty reduction capacity, but extends it by demonstrating that richness is not solely a media property. Instead, richness perception emerges from the interaction between media affordance and user interpretive competence (Z. Wang, 2022). This aligns with recent studies in sports education, emphasizing that ‘qualitative differences in technology-based learning’ are determined by user literacy (Wicaksono-Ikhsan & Suherman-Wawan, 2023). Okuonghae (2025) noted that satisfaction with immersive experiences provided by VR/AR technology varies dramatically depending on a learner’s literacy level. The findings of this study suggest that learners’ ‘digital interpretive skills’—their ability to extract meaning from media—must precede the media’s inherent richness. This represents a reinterpretation of classical MRT hypotheses in modern digital environments.

Our findings support UTAUT and MRT, but more importantly they refine both theories in digital sports learning contexts. First, we extend UTAUT by showing that “digital-native” cohorts are not homogeneous; rather, digital literacy stratifies acceptance beliefs, implying that personal competence operates as a meaningful boundary condition for UTAUT mechanisms in skills-based learning. Second, we advance MRT by proposing a competence-contingent media richness account: the same digital medium can be experienced as richer or poorer depending on learners’ interpretive competence for multimodal cues and interactive feedback. This refinement suggests that future MRT applications in technology-mediated learning should model user competence explicitly when predicting perceived richness and related outcomes.

### 6.2. Practical Implications

Based on these research findings, the following practical recommendations can be applied to sports education.

The following implications should not be interpreted as immediate implementation requirements but as staged design principles contingent on institutional resources and digital infrastructure. In many sports education contexts, disparities in device access, instructor digital competence, and organizational support structures may constrain direct adoption. Therefore, the proposed strategies are intended as scalable guidelines: low-resource environments may prioritize basic usability support and mediated instruction, whereas high-resource environments may progressively integrate adaptive AI-based feedback systems. This perspective positions digital literacy not only as an individual capability but also as an ecosystem-dependent educational condition.

First, it may be beneficial to design a sports learning interface tailored to different literacy levels where institutional support permits. For example, specialization is required in fields such as AI-based personalized coaching systems. For learners with low digital literacy (the lower group), AI coaches should prioritize providing intuitive visual feedback over complex numerical data. However, for the high-literacy group, precise data, such as heart rate, acceleration, and technical statistics, must be provided through multiple channels to meet their performance expectations. Recent research indicates that AI coaching systems can enhance learner motivation by approximately 30%. However, this is only achievable when a personalized interface tailored to literacy levels is implemented (H. Li et al., 2025).

Second, it is necessary to enhance sports leaders’ digital coaching capabilities. Therefore, sports instructors should serve as ‘digital mentors’ who preemptively assess learners’ literacy levels and recommend suitable digital assistive tools. This suggests that the key to technology adoption lies not only in introducing technology but also in establishing a support system within the learning environment.

Third, an enabling environment was created to bridge the digital divide. Subgroups with low digital literacy perceived significant effort expectancies in using technology. Therefore, physical and systemic support systems must be strengthened, such as deploying dedicated technical support staff within sports facilities or attaching simplified manuals using QR codes to lower the psychological barriers to technology adoption.

Fourth, a media strategy centered on interaction between sports instructors and learners should be introduced. The immediacy and personalization of feedback play a crucial role, so when developing sports apps or platforms, it is essential to enhance ‘real-time interaction’ capabilities between learners and technology beyond simple information delivery. In particular, Generation Z values personalized experiences. Therefore, AI-based customized feedback systems powered by learning data can serve as a bridge to increasing engagement among learners with below-average digital literacy.

Taken together, the study does not merely confirm UTAUT and MRT but connects them through a competence-contingent mechanism. Digital literacy operates as a cognitive translation layer that determines whether technological affordances become acceptance beliefs (UTAUT) and whether communication affordances become perceived richness (MRT). This integrative interpretation provides a theoretical linkage between adoption cognition and media experience that prior studies have examined separately.

## 7. Conclusions

This study demonstrated that even among Generation Z, commonly regarded as digital natives, meaningful differences in technology acceptance and media richness perceptions in sports learning exist according to individual digital literacy levels. By integrating UTAUT and Media Richness Theory within a contemporary sports education context, the findings highlight digital literacy as a salient individual-level correlate rather than a generational constant. Learners with higher digital literacy exhibited more favorable evaluations of technology-related expectations and perceived richer media environments, underscoring the heterogeneous nature of technology use within the same generation. These results suggest that technology-driven sports education should move beyond uniform implementation strategies. Instead, learner-centered approaches—such as adaptive AI-based feedback systems and strengthened digital mentoring competencies among coaches—may help reduce digital exclusion and may enhance educational effectiveness.

## 8. Limitations and Future Research

This study has several limitations that warrant consideration in future research. First, the sample was limited to Generation Z participants in the Republic of Korea, which constrains the generalizability of the findings to other regions and cultural contexts. Comparative cross-cultural studies are therefore needed to examine whether similar patterns of sports media learning emerge across different societies. Second, as the study relied on self-reported survey data, future research should incorporate behavioral observations, experimental designs, or log data analyses to capture actual technology use in sports learning environments. Third, although the analysis was not restricted to a specific sport, technology adoption may vary according to sport characteristics, such as individual versus team sports or indoor versus outdoor contexts. Fourth, experimental studies examining the effects of digital literacy-enhancing interventions on sports learning outcomes are required to establish clearer causal relationships. Finally, given the cross-sectional design, temporal ordering cannot be established; therefore, causal interpretations should be avoided and reverse causality remains possible. Future research should explore the nuanced relationship between digital literacy and learning outcomes by introducing control variables and longitudinal modeling, and verify whether the observed relationships translate into actual learning outcomes through longitudinal and behavioral data.

## Figures and Tables

**Table 1:** Descriptive statistics of survey respondents.

		Group 1	Group 2	Group 3
Digital Literacy Level		Low	Medium	High
Gender	Male	49 (56.3%)	49 (61.3%)	48 (68.6%)
	Female	38 (43.7%)	31 (38.7%)	22 (31.4%)
Education	High school graduation	10 (11.5%)	2 (2.5%)	5 (7.1%)
	Enrollment and graduation from a 2-year college	8 (9.2%)	-	2 (2.9%)
	University enrollment and graduation	55 (63.2%)	62 (77.5%)	39 (55.7%)
	Graduate school enrollment and graduation	14 (16.1%)	16 (20.0%)	24 (34.3%)
Participatory Sports	Martial Arts Sports	25 (28.8%)	22 (27.5%)	25 (35.7%)
	Fitness	15 (17.2%)	24 (30.0%)	14 (20.0%)
	Yoga & Pilates	10 (11.5%)	5 (6.3%)	4 (5.7%)
	Golf	5 (5.7%)	4 (5.0%)	8 (11.4%)
	Swimming	2 (2.3%)	-	1 (1.4%)
	Ball sports	14 (16.1%)	14 (17.5%)	7 (10.0%)
	Racket sports	9 (10.3%)	4 (5.0%)	4 (5.7%)
	Running	5 (5.7%)	4 (5.0%)	4 (5.7%)
	Extreme Sports	2 (2.4%)	3 (3.8%)	3 (4.4%)
Primary sports learning media used	YouTube	68 (78.3%)	56 (70.0%)	36 (51.4%)
	SNS	17 (19.5%)	22 (27.5%)	26 (37.1%)
	Online lecture platform	1 (1.1%)	-	6 (8.6%)
	Mobile app	1 (1.1%)	2 (2.5%)	2 (2.9%)
Main purpose of learning media	Physical fitness improvement	9 (10.3%)	5 (6.2%)	5 (7.1%)
	Sports Skill Acquisition	31 (35.7%)	36 (45.0%)	34 (48.6%)
	Hobbies and Leisure	29 (33.4%)	21 (26.3%)	16 (22.9%)
	Lose weight	17 (19.5%)	12 (15.0%)	11 (15.7%)
	Certification study	1 (1.1%)	6 (7.5%)	4 (5.7%)
Average time spent on sports learning	Less than 30 min	41 (47.1%)	36 (45.0%)	14 (20.0%)
	30 min to 1 h	34 (39.1%)	36 (45.0%)	37 (52.9%)
	1 to 2 h	8 (9.2%)	8 (10.0%)	10 (14.3%)
	over 2 h	4 (4.6%)	-	9 (12.8%)
Total		87 (100%)	80 (100%)	70 (100%)

Note. % = Amount in each hundred in each group.

**Table 2:** Result of the confirmatory factor analysis.

Factors	Items				*β*	S.E.	*t*	CR	AVE	ω	α
Digital literacy	I can analyze sports learning data using media or apps.				0.878	-	-	0.889	0.666	0.933	0.933
	I can effectively deliver sports-related information through digital platforms.				0.869	0.053	18.558				
	I am well aware of the importance of the process of analyzing and processing the information necessary for sports learning.				0.884	0.051	19.179				
	I understand the importance of information delivery and exchange in the sports learning process.				0.895	0.051	19.073				
Performance expectancy	I believe that learning sports through media helps improve my athletic skills.				0.885	-	-	0.908	0.712	0.941	0.940
	Using media allows you to learn sports skills more quickly.				0.878	0.053	19.452				
	Learning sports through media enhances my learning efficiency and performance.				0.912	0.046	21.170				
	Learning sports through media enhances my athletic abilities and increases my potential for achievement.				0.903	0.049	20.732				
Effort expectancy	The process of learning sports through media is clear and easy to understand.				0.842	-	-	0.895	0.680	0.926	0.924
	I can easily learn sports training methods using media.				0.923	0.056	18.953				
	Sports learning through media is generally easy to use.				0.862	0.058	16.888				
	Learning sports through media is not difficult for me.				0.855	0.065	16.652				
Social influence	People who influence me think I should learn sports by utilizing media.				0.848	-	-	0.890	0.670	0.945	0.944
	The people who matter to me view learning sports through media positively.				0.916	0.052	19.408				
	Those with higher skill levels than me recommend learning sports through media.				0.913	0.056	19.259				
	People around me generally support learning sports through media.				0.927	0.052	19.851				
Facilitating conditions	I have the resources needed to study online sports lectures.				0.893	-	-	0.868	0.623	0.941	0.941
	I possess the knowledge required to study online sports lectures.				0.945	0.044	23.640				
	The online sports lecture platform I use cannot be replaced by any other platform.				0.883	0.049	20.138				
	If you encounter problems while learning online sports lectures, there is someone (or a group) who can help you.				0.854	0.051	18.708				
Multiple channels	I can exchange information with instructors or others through sports media.				0.840	-	-	0.861	0.607	0.921	0.920
	I can understand the other person’s intentions through sports videos or coaching audio by analyzing their voice tone or intonation.				0.879	0.061	17.162				
	I can discuss physical movements (e.g., demonstration videos, posture expressions) through sports media.				0.860	0.061	16.566				
	I can discern the intentions of others through nonverbal expressions such as facial expressions and gestures in sports media.				0.871	0.066	16.900				
Immediacy of feedback	I can immediately see the reactions of others (coaches and teammates) while learning sports online.				0.941	-	-	0.916	0.732	0.963	0.962
	When I use sports media, my reactions are transmitted to the other person in real time.				0.921	0.036	26.758				
	I can quickly grasp my opponent’s opinions or feedback during sports training.				0.933	0.033	28.145				
	I can provide immediate feedback on sports instructors’ opinions in the media.				0.925	0.036	27.261				
Personalness	When I use sports learning media, I can feel the presence of coaches or teammates.				0.908	-	-	0.914	0.725	0.965	0.965
	I feel that sports learning media promotes social interaction and engagement.				0.941	0.040	25.549				
	I find sports learning media to be human and warm.				0.946	0.040	25.937				
	I feel a personal connection or sense of belonging through sports learning media.				0.945	0.040	25.861				
Model fit	χ^2^	*df*	*p*	NFI	IFI	TLI	CFI	SRMR		RMSEA	
	779.013	436	0.000	0.914	0.960	0.954	0.960	0.037		0.058	

**Table 3:** Descriptive statistics and correlations between key variables.

Classification	1	2	3	4	5	6	7	8
Digital literacy	**0.816**							
Performance expectancy	0.688 **	**0.844**						
Effort expectancy	0.651 **	0.750 **	**0.824**					
Social influence	0.560 **	0.594 **	0.603 **	**0.819**				
Facilitating conditions	0.538 **	0.491 **	0.501 **	0.606 **	**0.789**			
Multiple channels	0.617 **	0.582 **	0.580 **	0.616 **	0.556 **	**0.779**		
Immediacy of feedback	0.500 **	0.370 **	0.470 **	0.418 **	0.493 **	0.616 **	**0.856**	
Personalness	0.536 **	0.400 **	0.472 **	0.549 **	0.437 **	0.554 **	0.667 **	**0.852**
Mean	4.76	5.31	5.17	4.82	4.69	4.86	4.56	4.58
Standard Deviation	1.20	1.17	1.10	1.35	1.43	1.24	1.46	1.54
Skewness	−0.805	−0.715	−0.429	−0.218	−0.313	−0.618	−0.416	−0.308
Kurtosis	−0.145	0.067	−0.173	−0.537	−0.428	−0.132	−0.749	−0.492

Note. ** *p* < 0.01, Bold = $\sqrt{AVE}$.

**Table 4:** Results of multivariate analysis of variance.

Variables	Sub-Factors	*df*	F	*p*	*ηp* ^2^
UTAUT	Performance expectancy	2	64.150	<0.001	0.354
	Effort expectancy	2	60.529	<0.001	0.341
	Social influence	2	47.229	<0.001	0.288
	Facilitating conditions	2	42.412	<0.001	0.266
MRT	Multiple channels	2	55.647	<0.001	0.322
	Immediacy of feedback	2	40.364	<0.001	0.257
	Personalness	2	49.685	<0.001	0.298

Note. *p* < 0.001.

**Table 5:** Average Comparison by Group.

	a	b	c	d	e	f	g
G1	4.48	4.41	4.10	3.89	4.00	3.64	3.61
G2	5.46	5.29	4.68	4.65	5.07	4.84	4.66
G3	6.18	5.97	5.57	5.71	5.69	5.39	5.69

Note. G1 = Low digital literacy, G2 = Medium digital literacy, G3 = High digital literacy. a = Performance expectancy, b = Effort expectancy, c = Social influence, d = Facilitating conditions, e = Multiple channels, f = Immediacy of feedback, g = Personalness.

**Table 6:** Results of post hoc analysis.

		a	b	c	d	e	f	g
G1	G2	<0.001 ***	<0.001 ***	0.005 **	<0.001 ***	<0.001 ***	<0.001 ***	<0.001 ***
	G3	<0.001 ***	<0.001 ***	<0.001 ***	<0.001 ***	<0.001 ***	<0.001 ***	<0.001 ***
G2	G3	<0.001 ***	<0.001 ***	<0.001 ***	<0.001 ***	0.001 **	0.031 *	<0.001 ***

Note. G1 = Low digital literacy, G2 = Medium digital literacy, G3 = High digital literacy. a = Performance expectancy, b = Effort expectancy, c = Social influence, d = Facilitating conditions, e = Multiple channels, f = Immediacy of feedback, g = Personalness. *** *p* < 0.001, ** *p* < 0.01, * *p* < 0.05.

## Data Availability

The data supporting the findings of this study are available from the corresponding author upon reasonable request; however, they are not publicly accessible due to ethical constraints and the need to safeguard participants’ privacy.

## Abbreviations

The following abbreviations are used in this manuscript:

AI	Artificial Intelligence
AR	Augmented Reality
AVE	Average Variance Extracted
CFA	Confirmatory Factor Analysis
CFI	Comparative Fit Index
CR	construct reliability
EE	effort expectancy
FB	feedback
FC	facilitating conditions
ICT	information and communication technology
IFI	Incremental Fit Index
IRB	Institutional Review Board
MANOVA	Multivariate Analysis of Variance
MC	multiple channels
MRT	Media Richness Theory
NIA	National Information Society Agency
OECD	Organization for Economic Co-operation and Development
PE	performance expectancy
PS	personalness
RMSEA	Root Mean Square Error of Approximation
SI	social influence
SRMR	Standardized Root Mean Square Residual
TAM	Technology Acceptance Model
TLI	Tucker–Lewis Index
UTAUT	Unified Theory of Acceptance and Use of Technology
VR	Virtual Reality

## References

[B1-behavsci-16-00343] Al-Adwan A. S., Li N., Al-Adwan A., Abbasi G. A., Albelbisi N. A., Habibi A. (2023). Extending the technology acceptance model (TAM) to predict university students’ intentions to use metaverse-based learning platforms. Education and Information Technologies.

[B2-behavsci-16-00343] Alanoglu M., Karabatak S., Yang H. (2025). Understanding university students’ self-directed online learning in the context of emergency remote teaching: The role of online learning readiness and digital literacy. Journal of Computing in Higher Education.

[B3-behavsci-16-00343] Alblooshi S., Abdul Hamid N. A. B. (2022). The effect of performance expectancy on actual use of e-learning throughout the mediation role of behaviour intention. Journal of E-Learning and Higher Education.

[B4-behavsci-16-00343] Alshammari S. H., Alkhwaldi A. F. (2025). An integrated approach using social support theory and technology acceptance model to investigate the sustainable use of digital learning technologies. Scientific Reports.

[B5-behavsci-16-00343] Ambarwati R., Harja Y. D., Thamrin S. (2020). The role of facilitating conditions and user habits: A case of Indonesian online learning platform. The Journal of Asian Finance, Economics and Business.

[B6-behavsci-16-00343] Anderson J. C., Gerbing D. W. (1988). Structural equation modeling in practice: A review and recommended two-step approach. Psychological Bulletin.

[B7-behavsci-16-00343] Battelle for Kids (2019). Framework for 21st century learning: P21 framework definitions. Partnership for 21st Century learning.

[B8-behavsci-16-00343] Cai X., Xian Y., Liu T., Zhou Y., Chen Q., Cui H. (2025). A study on factors influencing digital sports participation among Chinese secondary school students based on explainable machine learning. Scientific Reports.

[B9-behavsci-16-00343] Camilleri M. A. (2024). Factors affecting performance expectancy and intentions to use ChatGPT: Using SmartPLS to advance an information technology acceptance framework. Technological Forecasting and Social Change.

[B10-behavsci-16-00343] Chen J., Liu M., Hu J., Zhang J. (2025). The impact of health communication through sports social media on adolescents’ sport participation attitudes: A moderated mediation model. Scientific Reports.

[B11-behavsci-16-00343] Cho A. Y., Park I. W., Ko Y. J. (2019). Exploring the mediation effect of flow on the effects of digital literacy on satisfaction and achievement in smartpad; based math classes. The Journal of Research in Education.

[B12-behavsci-16-00343] Cossich V. R., Carlgren D., Holash R. J., Katz L. (2023). Technological breakthroughs in sport: Current practice and future potential of artificial intelligence, virtual reality, augmented reality, and modern data visualization in performance analysis. Applied Sciences.

[B13-behavsci-16-00343] Daft R. L., Lengel R. H. (1986). Organizational information requirements, media richness and structural design. Management Science.

[B14-behavsci-16-00343] Davis F. D. (1989). Perceived usefulness, perceived ease of use, and user ac-ceptance of information technology. MIS Quarterly.

[B15-behavsci-16-00343] Deng H., Tao Y. (2025). Digital transformation of physical education teaching in higher education based on the OMO framework. Curriculum and Teaching Methodology.

[B16-behavsci-16-00343] Ferry D. L., Kydd C. T., Sawyer J. E. (2001). Measuring Facts of Media Richness. Journal of Computer Information Systems.

[B17-behavsci-16-00343] Fornell C., Larcker D. F. (1981). Evaluating structural equation models with unobservable variables and measurement error. Journal of Marketing Research.

[B18-behavsci-16-00343] Frandsen K. (2019). Sport and mediatization.

[B19-behavsci-16-00343] Getenet S., Cantle R., Redmond P., Albion P. (2024). Students’ digital technology attitude, literacy and self-efficacy and their effect on online learning engagement. International Journal of Educational Technology in Higher Education.

[B20-behavsci-16-00343] Gilster P. (1997). Digital literacy.

[B21-behavsci-16-00343] Goo K. B. (2023). The development of sports content in the fourth industrial revolution. Philosophy of Movement: The Journal of the Korean Society for the Philosophy of Sport, Dance, and Martial Arts.

[B22-behavsci-16-00343] Gottschalk F., Weise C. (2023). Digital equity and inclusion in education: An overview of practice and policy in OECD countries.

[B23-behavsci-16-00343] Hu X., Chen X., Davison R. M. (2019). Social support, source credibility, social influence, and impulsive purchase behavior in social commerce. International Journal of Electronic Commerce.

[B24-behavsci-16-00343] Kabakus A. K., Bahcekapili E., Ayaz A. (2025). The effect of digital literacy on technology acceptance: An evaluation on administrative staff in higher education. Journal of Information Science.

[B25-behavsci-16-00343] Ke Q., Gong Y., Ke C. (2025). Bridging AI literacy and UTAUT constructs: Structural equation modeling of AI adoption among Chinese university students. Humanities and Social Sciences Communications.

[B26-behavsci-16-00343] Kline R. B. (1998). Software review: Software programs for structural equation modeling: Amos, EQS, and LISREL. Journal of Psychoeducational Assessment.

[B27-behavsci-16-00343] Ko M. S. (2020). Sports education research and practice in preparation for future educational environment changes. Korean Journal of Sport Pedagogy.

[B28-behavsci-16-00343] Lee Y. H., Ryu M. H. (2019). Study on use intention of Chinese unmanned convenience stores by applying UTAUT and theory of experience economy: Verifying the moderating effect of reliability. Journal of Distribution and Management Research.

[B29-behavsci-16-00343] Li H., Yang Z., Li J. (2025). The impact of digital literacy on individual health: A perspective based on fitness exercise. Frontiers in Public Health.

[B30-behavsci-16-00343] Li Z., Slavkova O., Gao Y. (2022). Role of digitalization, digital competence, and parental support on performance of sports education in low-income college students. Frontiers in Psychology.

[B31-behavsci-16-00343] Lin Q., Yin Y., Tang X., Hadad R., Zhai X. (2020). Assessing learning in technology-rich maker activities: A systematic review of empirical research. Computers & Education.

[B32-behavsci-16-00343] Liu X., Wang J., Luo Y. (2025). Acceptance and use of technology on digital learning resource utilization and digital literacy among Chinese engineering students: A longitudinal study based on the UTAUT2 model. Behavioral Sciences.

[B33-behavsci-16-00343] MacCallum R. C., Zhang S., Preacher K. J., Rucker D. D. (2002). On the practice of dichotomization of quantitative variables. Psychological Methods.

[B34-behavsci-16-00343] Mammadov R. (2022). Media choice in times of uncertainty—Media richness theory in context of media choice in times of political and economic crisis. Advances in Journalism and Communication.

[B35-behavsci-16-00343] Martins C., Oliveira T., Popovič A. (2014). Understanding the internet banking adoption: A unified theory of acceptance and use of technology and perceived risk application. International Journal of Information Management.

[B36-behavsci-16-00343] Martín-Rodríguez A., Madrigal-Cerezo R. (2025). Technology-enhanced pedagogy in physical education: Bridging engagement, learning, and lifelong activity. Education Sciences.

[B37-behavsci-16-00343] McDonald R. P. (1999). Test theory: A unified treatment.

[B38-behavsci-16-00343] Meng Y., Xu W., Liu Z., Yu Z. G. (2024). Scientometric analyses of digital inequity in education: Problems and solutions. Humanities and Social Sciences Communications.

[B39-behavsci-16-00343] Ministry of Science and ICT, National Information Society Agency (2025). 2024 digital divide survey.

[B40-behavsci-16-00343] Mutz M., Müller J., Reimers A. K. (2021). Use of digital media for home-based sports activities during the COVID-19 pandemic: Results from the German SPOVID survey. International Journal of Environmental Research and Public Health.

[B41-behavsci-16-00343] Nikou S., De Reuver M., Mahboob Kanafi M. (2022). Workplace literacy skills—How information and digital literacy affect adoption of digital technology. Journal of Documentation.

[B42-behavsci-16-00343] Nunnally J. C., Bernstein I. H. (1994). The Assessment of Reliability. Psychometric Theory.

[B43-behavsci-16-00343] OECD (n.d.). The OECD learning compass 2030.

[B44-behavsci-16-00343] OECD (2019). PISA 2018 results (Volume I): What students know and can do.

[B45-behavsci-16-00343] Okuonghae O. (2025). Advancing information literacy through immersive technologies in the metaverse age: A rapid review. Digital Library Perspectives.

[B46-behavsci-16-00343] Oregon E., McCoy L., Carmon-Johnson L. (2018). Case analysis: Exploring the application of using rich media technologies and social presence to decrease attrition in an online graduate program. Journal of Educators Online.

[B47-behavsci-16-00343] Osler L., Zahavi D. (2023). Sociality and embodiment: Online communication during and after COVID-19. Foundations of Science.

[B48-behavsci-16-00343] Özsari A., Tek T., Uysal H., Genç M., Toros T., Pepe Ş., Altin M. (2025). The effect of digital literacy on mental toughness: Research on a sport branch. Frontiers in Psychology.

[B49-behavsci-16-00343] Park E. Y. (2025). Factors related to digital literacy in people with disabilities: Focus on self-efficacy and attitude toward digital devices and technology. International Review of Economics & Finance.

[B50-behavsci-16-00343] Penalver-Andres J., Buetler K. A., Koenig T., Müri R. M., Marchal-Crespo L. (2021). Providing task instructions during motor training enhances performance and modulates attentional brain networks. Frontiers in Neuroscience.

[B51-behavsci-16-00343] Peng D., Yu Z. (2022). A literature review of digital literacy over two decades. Education Research International.

[B52-behavsci-16-00343] Preacher K. J., Rucker D. D., MacCallum R. C., Nicewander W. A. (2005). Use of the extreme groups approach: A critical reexamination and new recommendations. Psychological Methods.

[B53-behavsci-16-00343] Rasheed R. A., Kamsin A., Abdullah N. A. (2020). Challenges in the online component of blended learning: A systematic review. Computers & Education.

[B54-behavsci-16-00343] Reid L., Button D., Brommeyer M. (2023). Challenging the myth of the digital native: A narrative review. Nursing Reports.

[B55-behavsci-16-00343] Richardson J. T. (2011). Eta squared and partial eta squared as measures of effect size in educational research. Educational Research Review.

[B56-behavsci-16-00343] Rosli M. S., Saleh N. S. (2023). Technology enhanced learning acceptance among university students during COVID-19: Integrating the full spectrum of Self-Determination Theory and self-efficacy into the Technology Acceptance Model. Current Psychology.

[B57-behavsci-16-00343] Sannes D., Kim W. (2018). A theoretical framework of sports team’s well-being: An integrative perspective of emotional intelligence and equivocality on trust and happiness. Atlantic Marketing Journal.

[B58-behavsci-16-00343] Shadish W. R., Cook T. D., Campbell D. T. (2002). Experimental and quasi-experimental designs for generalized causal inference.

[B59-behavsci-16-00343] Soeprijanto S., Diamah A., Rusmono R. (2022). The effect of digital literacy, self-awareness, and career planning on engineering and vocational teacher education students’ learning achievement. JOTSE.

[B60-behavsci-16-00343] Soyer F., Güngör N. B., Soyer A. (2023). The effect of digital literacy levels of sports sciences faculty students on e-learning attitude. Journal of ROL Sport Sciences.

[B61-behavsci-16-00343] Stefán F., Ciesielski M., Weber A., Choromański K., Gotlib D., Taczanowska K. (2025). Understanding generational differences in digital skills and recreational behaviour for effective visitor management in forest destinations. Scientific Reports.

[B62-behavsci-16-00343] Su P. Y., Su C. H., Wang C. M., Fan K. K. (2024). Integrating media richness theory and technology acceptance model to study the learning outcomes of air quality education app. Journal of Internet Technology.

[B63-behavsci-16-00343] Swim N., Presley R., Thompson E. (2023). Digital development and technology in sport: A course to improve digital literacy in the sport management curriculum. Sport Management Education Journal.

[B64-behavsci-16-00343] Tang S., Geng J. (2025). Communication media and linguistic concreteness: Evidence from firm site visits in China. China Journal of Accounting Research.

[B65-behavsci-16-00343] Teo T. (2019). Students and teachers’ intention to use technology: Assessing their measurement equivalence and structural invariance. Journal of Educational Computing Research.

[B66-behavsci-16-00343] Turhan F. H. (2023). Perceptions of the effect of digital literacy levels of who take sports education students on e-learning. International e-Journal of Educational Studies.

[B67-behavsci-16-00343] Venkatesh V., Morris M. G., Davis G. B., Davis F. D. (2003). User acceptance of information technology: Toward a unified view. MIS Quarterly.

[B68-behavsci-16-00343] Wang C., Chen X., Yu T., Liu Y., Jing Y. (2024). Education reform and change driven by digital technology: A bibliometric study from a global perspective. Humanities and Social Sciences Communications.

[B69-behavsci-16-00343] Wang F., Ni X., Zhang M., Zhang J. (2024). Educational digital inequality: A meta-analysis of the relationship between digital device use and academic performance in adolescents. Computers & Education.

[B70-behavsci-16-00343] Wang Z. (2022). Media richness and continuance intention to online learning platforms: The mediating role of social presence and the moderating role of need for cognition. Frontiers in Psychology.

[B71-behavsci-16-00343] Wicaksono-Ikhsan N., Suherman-Wawan S. (2023). Digital literacy in physical education: A literature review. International Journal of Physical Education, Sports and Health.

[B72-behavsci-16-00343] Yu H. S. (2025). Student flow by lecture type (offline vs. online): Serial multiple mediation effects of perceived media richness and social presence. The Journal of Educational Information and Media.

[B73-behavsci-16-00343] Yu T. K., Lin M. L., Liao Y. K. (2017). Understanding factors influencing information communication technology adoption behavior: The moderators of information literacy and digital skills. Computers in Human Behavior.

[B74-behavsci-16-00343] Zakir S., Hoque M. E., Susanto P., Nisaa V., Alam M. K., Khatimah H., Mulyani E. (2025). Digital literacy and academic performance: The mediating roles of digital informal learning, self-efficacy, and students’ digital competence. Frontiers in Education.

